# Endobronchial closure of the bronchopleural fistula with the ventricular septal defect occluder: a case series

**DOI:** 10.1186/s12890-021-01676-3

**Published:** 2021-10-07

**Authors:** Yang Bai, Yishi Li, Jing Chi, Shuliang Guo

**Affiliations:** Department of Respiratory and Critical Care Medicine, The First Affiliated Hospital of Chongqing Medical University, No.1 Youyi Road, Yuzhong District, Chongqing, 400016 PR China

**Keywords:** Bronchopleural fistula, Ventricular septal defect, Occluder, Bronchoscopic treatment, Case series

## Abstract

**Objectives:**

The ventricular septal defect (VSD) occluder has been reported to be a novel method for the closure of bronchopleural fistula (BPF). Our study was to confirm the use of VSD occluder in treating BPF after pneumonectomy or lobectomy.

**Methods:**

We performed a single-center, retrospective study of 10 consecutive patients (8 men and 2 women aged 29–70 years) with postoperative BPF receiving the VSD occluder treatment. We used the HeartR™ Membranous VSD occluder (Lifetech Scientific Co., Shenzhen, China) for the closure of BPF through flexible bronchoscopy under general anesthesia. Demographic characteristics, BPF characteristics, and clinical outcomes were collected from patients’ files using the standardized data abstraction forms.

**Results:**

The underlying diseases were lung cancer in 6 patients, pulmonary tuberculosis in 3, and bronchiectasis in 1. Right-sided BPFs occurred in 6 patients, and left-sided BPFs occurred in 4. Five patients were underweight with a body mass index < 18.5 kg/m^2^. The VSD was placed in all 10 patients with a 100% technical success rate and a 70% complete closure rate during follow-up with no complications, on a median follow-up period of 115 days (range 46–975 days). In 1 patient, the VSD occluder was reinstalled with complete closure; in 1 and 2 patients with underweight and chronic empyema, the VSD occluders partially and completely failed with good physical tolerance, respectively.

**Conclusions:**

Our study demonstrated the bronchoscopic closure of BPF after lung resection using the VSD occluder is an off-label but safe and effective method. We prefer to stabilize the BPF by eradicating the underlying diseases and providing nutritional support to those receiving VSD occluder closure treatment.

## Introduction

The bronchopleural fistula (BPF) is an uncommon but major complication of lung resection, associated with high mortality, especially in lung cancer patients [1]. Most patients with postpneumonectomy BPF are debilitated and have chronic empyema, leading to high morbidity [2]. Open thoracotomy or video-assisted thoracoscopic surgery is recommended for BPF closure if the patients could tolerate a major reoperation [3]. The success rate of surgery for BPF closure has been over 85 % in those patients [4, 5]. In some severely ill patients, the bronchoscopic treatment seems to be a well-tolerated and cost-effective procedure [6]. Various bronchoscopic treatments have been reported in small studies and case reports for BPF closure, such as the Amplatzer vascular occluder [7], one-way endobronchial valve [8], vascular occlusion coil [9], covered expandable metallic stent [10], adhesive tissue and fibrin glue [11], sclerosing agent [12], and et al.

The Amplatzer vascular occluder, designed to close the ventricular septal defect (VSD), was first used for endobronchial closure of BPF under general anesthesia in 2008 by Kramer and his colleagues [7]. Since then, the Amplatzer vascular occluder has been used increasingly and successfully in selected BPF patients worldwide [13–15]. In the most extensive case series (31 patients), improvement or resolution of BPF occurred in 96 % of patients treated with the Amplatzer vascular occluder, lasting for up to 18 months [16]. This study aims to present our experience in treating postpneumonectomy BPF with the HeartR™ Membranous VSD occluder (Lifetech Scientific Co., Shenzhen, China). We have presented our data with an emphasis on long-term outcomes, especially in those with underweight or chronic empyema.

## Materials and methods

We performed a single-center, retrospective study of patients with BPF after pneumonectomy or lobectomy who presented to the First Affiliated Hospital of Chongqing Medical University in Chongqing, China. We reviewed the medical records, laboratory results, chest computed tomography images, bronchoscopy reports, and pathological findings for patients with BPF between January 2018 and March 2021. Patients with confirmed BPF receiving the implantation of VSD occluder were included in this study. The attempt to close the BPF with vascular occlusion coil and adhesive tissue in two patients failed before attending our department. All patients treated with the VSD occluder on a compassionate basis were not candidates for surgical repair and refused other treatment modalities due to financial burden. Written informed consent was obtained from all patients, including the off-label use of the VSD occluder in the closure of BPF. We used the standardized data abstraction forms to gather information on demographic characteristics, BPF characteristics (etiology, location, long axis length, et al.), and clinical outcomes. The institutional scientific committee approved the publication of this retrospective study.

**Fig. 1:**
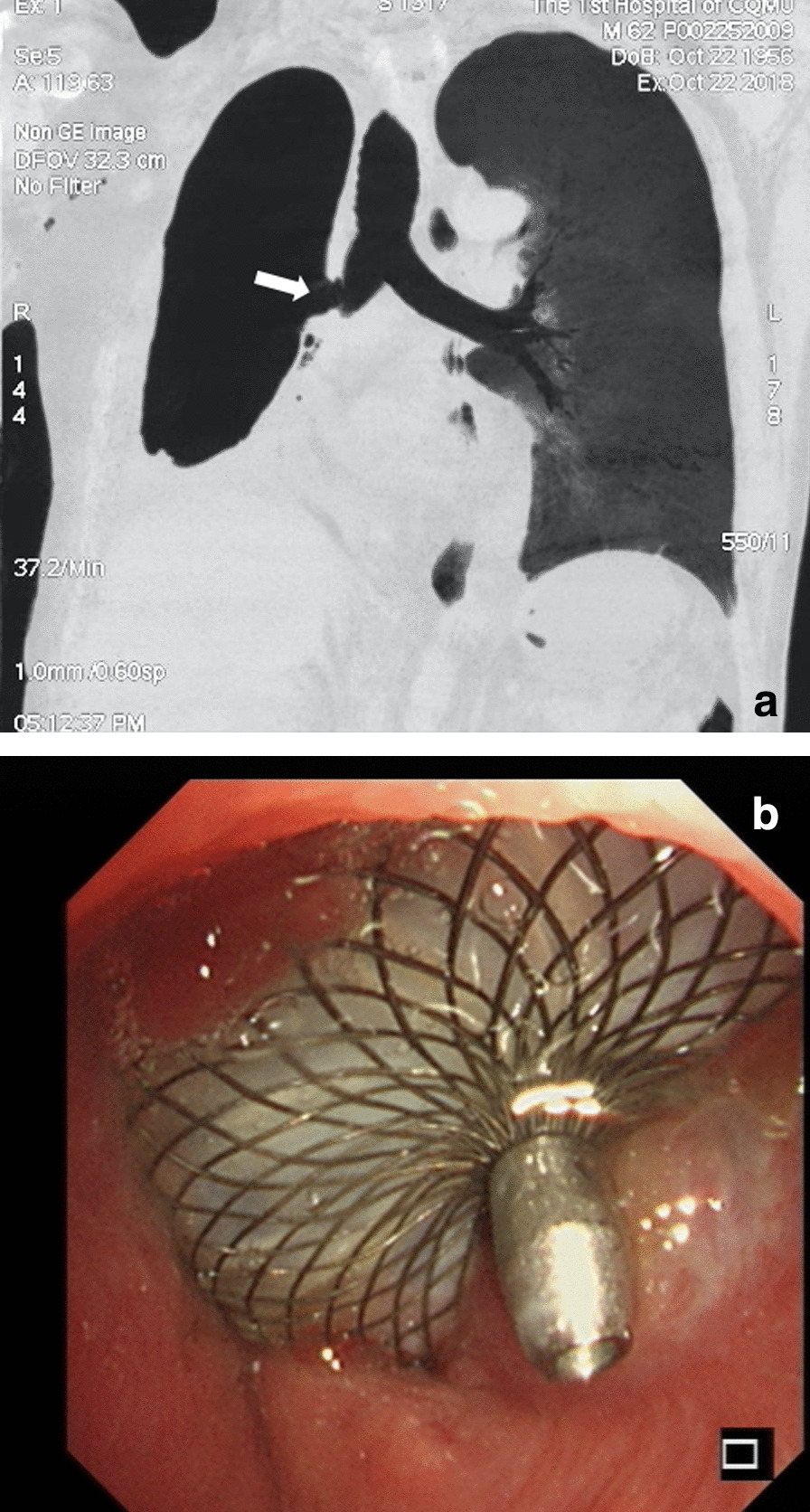
Patient no. 4 in Table 1. **a** Measuring the size of the BPF (white arrow) localized in the right middle bronchus via three-dimensional reconstruction of chest computed tomography images. **b** The bronchoscopic view of the large central BPF located in the right main bronchus completely closed by the VSD occluder with surrounding granulation tissue

We used the HeartR™ Membranous VSD occluder (Lifetech Scientific Co., Shenzhen, China), made of multi-layered nitinol wire mesh with low-profile retention disks. The central waist diameter ranges from 4 to 24 mm, with a fixed length of 3 mm. The symmetric disk diameter is 4–5 mm larger than the central waist diameter, giving an anchoring lip of 2–2.5 mm circumferentially. The central waist is anchored and stabilized into the fistula by the two symmetric disks. The polytetrafluoroethylene membrane in the VSD occluder increases the closing ability and reduces the inflow of purulent pleural effusion. Two independent specialized radiologists measured the long axis length of BPF using the three-dimensional reconstruction of chest computed tomography images (Fig. 1a). The selected VSD colluder’s central waist diameter was 4 mm larger than the measured long axis length of BPF, giving the occluder good compression strength. The guidewire fixed with an occluder was inserted into the BPF through the bronchoscope work channel under general anesthesia. The proximal disk of the occluder was unfolded and positioned directly under flexible bronchoscopic observation. The guidewire was disconnected from the occluder when it was positioned correctly. The immediate technical success was defined as complete closure of the BPF using VSD occluders with an immediate absence of air leakage through the chest tube drainage. Complete closure (assessed at every visit) was defined as BPF completely closed with the VSD occluder, and incomplete closure was BPF mostly closed with previous symptoms completely resolved, while failure was BPF not closed with the persistence of symptoms.

**Table 1:** Demographic and clinical data of patients with postoperative bronchopleural fistula treated by ventricular septal defect occluder

Patient no.	Age/sex	BMI (kg/m^2^)	Underlying diseases	Chronic empyema	BPF location	BPF size (mm)	Operation time (min)	Long-term outcomes
1	29/M	17.8	TB	Yes	RUL	6	48	Incomplete closure
2	65/M	16.2	NSCLC	No	LMB	4	45	Complete closure
3	55/M	20.5	TB	Yes	RUL	8	40	Complete closure
4	62/M	19.8	NSCLC	Yes	RMB	8	35	Complete closure
5	36/M	15.0	TB	Yes	RUL	6	40	Failure
6	70/M	17.7	NSCLC	No	LUL	4	30	Complete closure
7	57/F	25.3	CF	Yes	LLL	5	55	Complete closure
8	60/M	17.7	NSCLC	Yes	RUL	2	56	Failure
9	52/M	21.9	NSCLC	Yes	RUL	8	33	Complete closure
10	56/F	22.7	NSCLC	Yes	LMB	6	29	Complete closure

*BPF* Bronchopleural fistula, *M* male, *F* female, *TB* tuberculosis, *NSCLC* non-small cell lung cancer, *CF* cystic fibrosis, *RUL* right upper lobe, *LMB* left main bronchus, *RMB* right main bronchus, *LUL* left upper lobe, *LLL* left lower lobe

## Results

A total of ten consecutive patients with post-pneumonectomy or post-lobectomy BPF receiving the insertion of HeartR™ Membranous VSD occluder were recruited in this study (Table 1). The male/female ratio was 8/2, with ages ranging from 29 to 70 years. Five patients were underweight with a body mass index (BMI) below 18.5 kg/m^2^. Eight patients had chronic empyema associated with BPF. Non-small cell lung cancer was the underlying disease in 6 patients, pulmonary tuberculosis in 3 patients, and cystic fibrosis in 1 patient. The locations of BPF were right upper lobe in 5 patients, right main stem bronchus in 1 patient, left main stem bronchus in 2 patients, left upper lobe in 1 patient, and left lower lobe in 1 patient. All patients with BPF were not recommended the primary surgical repair due to underweight or chronic empyema.

**Fig. 2:**
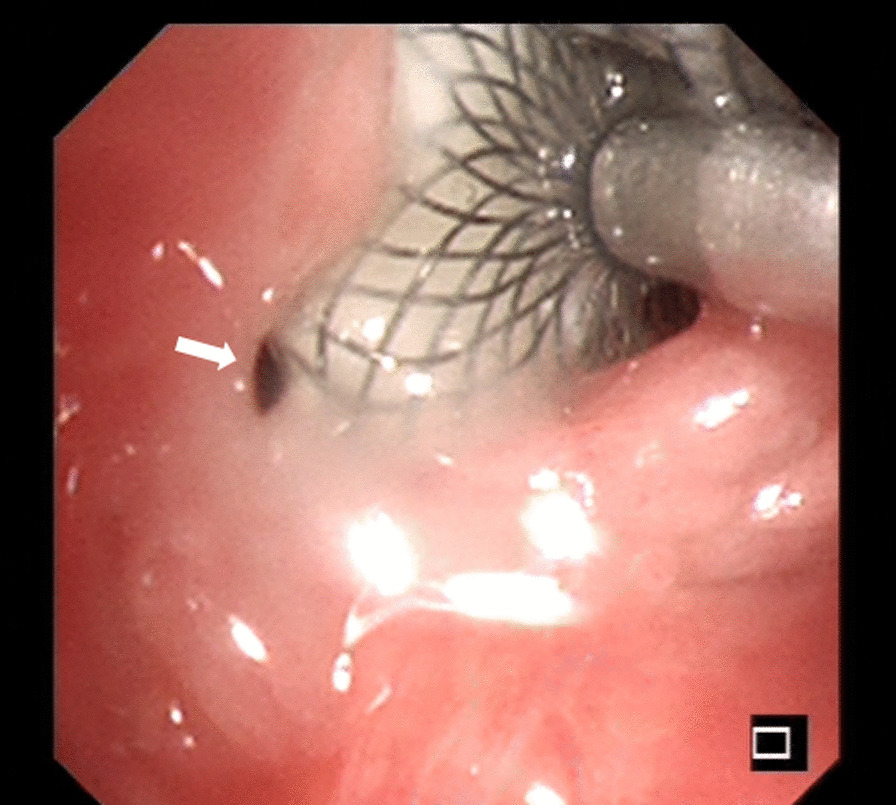
Patient no. 1 in Table 1. The bronchoscopic view of the large central BPF in the right upper lobe incompletely covered (white arrow) by the VSD occluder

**Fig. 3:**
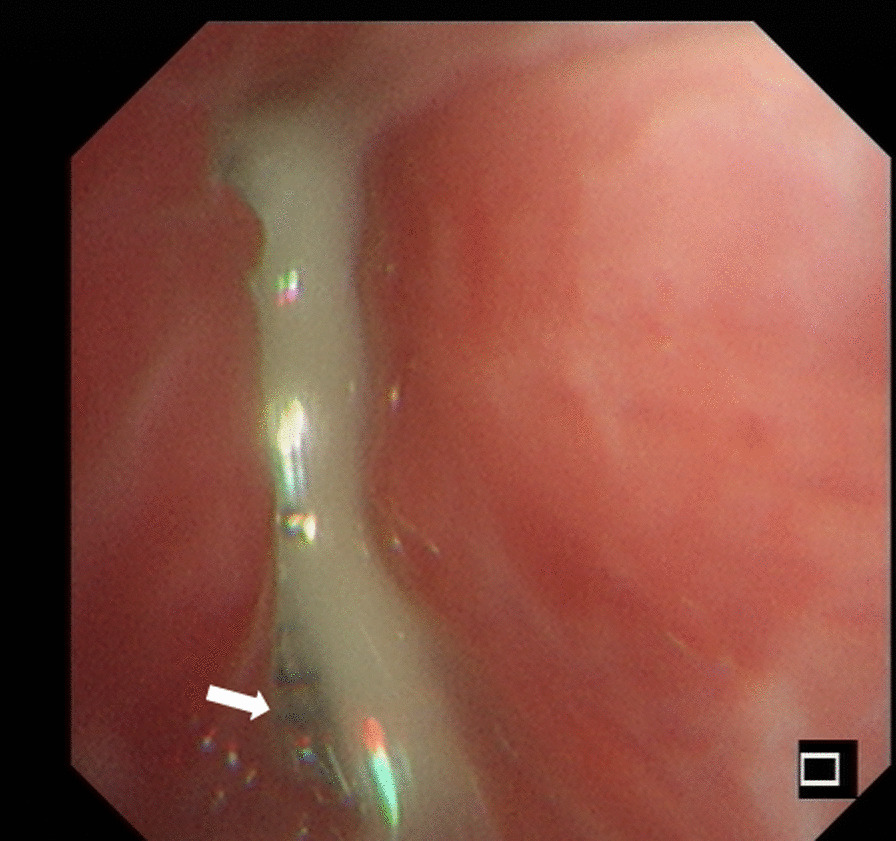
Patient no. 5 in Table 1. The bronchoscopic view of the large central BPF in the right upper lobe partially covered (white arrow) by the VSD occluder with purulent discharge

**Fig. 4:**
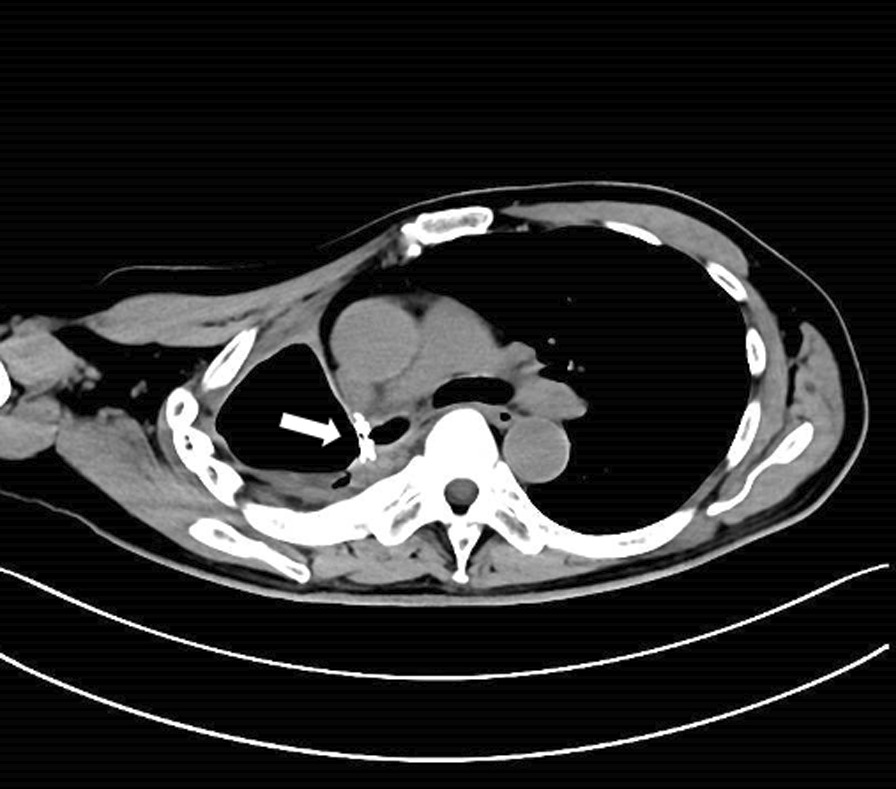
Patient no. 4 in Table 1. The BPF in the right main bronchus was completely closed by the VSD occluder (white arrow) in the latest chest computed tomography

The immediate technical success rate of the HeartR™ Membranous VSD occluder was 100 % for the closure of BPFs with long axis lengths of about 2-8mm in this study. The median operation time was 40 min (range 29–55 min). Symptoms of BPF, such as cough with purulent sputum and persistent air leakage through the chest tube drainage, disappeared just after the insertion of the VSD occluder. This led to the granulation tissue formation around the HeartR™ Membranous VSD occluder for the closure of BPF in seven patients, which was confirmed by bronchoscopy, on a median follow-up period of 115 days (range 46–975 days). In patient no. 4, the BPF located in the right main bronchus was completely closed by the VSD occluder with surrounding granulation tissue (Fig. 1b). In patient no. 6, the VSD occluder was dislocated 2 months after the insertion and then repositioned into the BPF through bronchoscopy under general anesthesia, and the BPF was successfully closed in the follow-up. Incomplete closure and failure with the HeartR™ Membranous VSD occluder occurred in the other three patients. In patient no. 1, incomplete closure occurred 32 months after the insertion. The VSD occluder was not replaced because the patient’s symptoms improved significantly, and the disclosure was minimal (Fig. 2). In patient no. 5 (Fig. 3) and no. 8, the VSD occluder failed to close the BPF. They refused to replace a new VSD occluder due to relatively good physical tolerance and its associated financial burden. No complications or adverse events were observed during and after the implantation of VSD HeartR™ Membranous occluder. The BPF in the right main bronchus was completely closed by the VSD occluder in the latest chest computed tomography of patient no.4 (Fig. 4). All patients exhibited no signs of residual or recurrence of underlying diseases in the follow-up

## Discussion

The VSD occluder was placed in all patients with BPF after lung resection with a 100 % immediate technical success rate and a 70 % complete closure rate during follow-up in our study. The VSD occluder closure rate was in consistent with other vascular occluder studies in the closure of BPF [16, 17]. Nine patients with BPF after lung resection were with a BMI lower than 25 kg/m^2^ in this study, and two failures with the VSD occluder occurred in patients with a BMI lower than 18.5 kg/m^2^. Patients with a BMI lower than 25 kg/m^2^ are at increased risk of BPF after lung resection [18, 19]. The initial procedure in patients with empyema after pneumonectomy or lobectomy is to identify BPF using a flexible bronchoscope, especially in those with a BMI lower than 25 kg/m^2^. The underweight might be a risk factor for the failure of closing BPF with the vascular occluder. The underweight or malnutrition might result in the enlargement of the BPF and the VSD occluder’s dislocation into the pleural cavity.

Surgical resection and closure of BPF combined with antibiotics, serial debridement, chest drainage, and obliteration of the residual pleural space are recommended if the clinical situation allows a major reoperation [20–22]. The bronchoscopic treatment has evolved as an adjuvant or complementary procedure to surgery, enabling the BPF visualization (location and size), bronchial stump evaluation, and treatment strategy decision simultaneously, particularly in those severely ill patients [6]. The initial bronchoscopic treatment of BPF depends heavily on the size of the fistula. Primary bronchoscopic treatments in managing large BPF include surgery [3], VSD occluder [10], and Y-type covered metallic expandable stent [16]. All patients with postoperative BPF included in our study were refractory cases who failed antibiotics, chest tube drainage, tube thoracostomy, and were not candidates for surgical repair because of underweight or chronic empyema. They also refused the Y-type covered metallic expandable stent due to financial burden and complications such as stent intolerance, mucus retention, pneumonia, and stent restenosis. Other bronchoscopic treatments have been reported in closing small BPF, such as adhesive tissue and fibrin glue, sclerosing agent, one-way endobronchial valve, endobronchial Watanabe spigot, vascular occlusion coil, and mesenchymal stem cells, some initial attempts of which had failed in our patients. Fibrin glue and vascular occlusion coil did not close the BPF with oracle about 2 mm in patient no.2. The VSD HeartR™ Membranous occluder available from 4 to 24 mm could be feasibly applied to close BPF with different sizes. We noticed that the VSD occluder used in our study had only a 3 mm waist length, which might not suit the BPF with a long tract. Other kinds of VSD or Amplatzer vascular occluders with longer waist lengths to accommodate the thick wall and long tract of BPF are commercially available.

VSD occluder can be positioned into the BPF with more accessible and faster manipulation through bronchoscope due to its flexible profile and small guidewire. Besides, the VSD occluder can minimize the clamping force to the bronchial stump and the radial stress on the BPF rim due to its multi-layered nitinol wire mesh with low-profile retention disks. BPFs > 8mm or large central BPFs are usually unsuitable for bronchoscopic treatment, such as adhesive tissue and fibrin glue [6, 23]. The bronchopleural air leak greater than 500 mL/breath during mechanical ventilation is associated with bronchoscopic treatment failure [23, 24]. The successful treatment of BPF in our study might also be due to the fistula being blocked by the polytetrafluoroethylene membrane in the VSD occluder, reducing air leak and promoting granulation tissue formation. On top of that, we carefully ruled out the residual or recurrence of underlying diseases, which could cause the failure of bronchoscopic closure of BPF with VSD occluder [25].

## Conclusions

We reported a case series of endobronchial closure of BPF using the VSD occluder after lung resection. Our preference is to stabilize the BPF by eradicating the underlying diseases and providing nutritional support to those receiving VSD occluder closure treatment. The bronchoscopic treatment with VSD occluder, an off-label but safe and effective method, is worth trying in selected cases for the closure of postoperative BPF. More studies are needed to assure the VSD occluder in the treatment of postoperative BPF.

## Data Availability

The datasets used and analyzed during the current study are available from the corresponding author on reasonable request.

## References

[1] Li S, Fan J, Liu J (2016). Neoadjuvant therapy and risk of bronchopleural fistula after lung cancer surgery: a systematic meta-analysis of 14 912 patients. Jpn J Clin Oncol..

[2] Sirbu H, Busch T, Aleksic I, Schreiner W, Oster O, Dalichau H (2001). Bronchopleural fistula in the surgery of non-small cell lung cancer: incidence, risk factors, and management. Ann Thorac Cardiovasc Surg..

[3] Shen KR, Bribriesco A, Crabtree T (2017). The American Association for Thoracic Surgery consensus guidelines for the management of empyema. J Thorac Cardiovasc Surg..

[4] Puskas JD, Mathisen DJ, Grillo HC, Wain JC, Wright CD, Moncure AC (1995). Treatment strategies for bronchopleural fistula. J Thorac Cardiovasc Surg..

[5] Massera F, Robustellini M, Pona CD, Rossi G, Rizzi A, Rocco G (2006). Predictors of successful closure of open window thoracostomy for postpneumonectomy empyema. Ann Thorac Surg..

[6] Lois M, Noppen M (2005). Bronchopleural fistulas: an overview of the problem with special focus on endoscopic management. Chest..

[7] Kramer MR, Peled N, Shitrit D (2008). Use of Amplatzer device for endobronchial closure of bronchopleural fistulas. Chest..

[8] Travaline JM, McKenna RJ, De Giacomo T (2009). Treatment of persistent pulmonary air leaks using endobronchial valves. Chest..

[9] Sivrikoz CM, Kaya T, Tulay CM, Ak I, Bilir A, Döner E (2007). Effective approach for the treatment of bronchopleural fistula: application of endovascular metallic ring-shaped coil in combination with fibrin glue. Ann Thorac Surg..

[10] Han X, Yin M, Li L (2018). Customized airway stenting for bronchopleural fistula after pulmonary resection by interventional technique: single-center study of 148 consecutive patients. Surg Endosc..

[11] Eng J, Sabanathan S (1990). Successful closure of bronchopleural fistula with adhesive tissue. Case report. Scand J Thorac Cardiovasc Surg..

[12] Boudaya MS, Smadhi H, Zribi H (2013). Conservative management of postoperative bronchopleural fistulas. J Thorac Cardiovasc Surg..

[13] Tedde ML, Scordamaglio PR, Minamoto H, Figueiredo VR, Pedra CC, Jatene FB (2009). Endobronchial closure of total bronchopleural fistula with Occlutech Figulla ASD N device. Ann Thorac Surg..

[14] Gulkarov I, Paul S, Altorki NK, Lee PC (2009). Use of Amplatzer device for endobronchial closure of bronchopleural fistulas. Interact Cardiovasc Thorac Surg..

[15] Yang L, Kong J, Tao W (2013). Tuberculosis bronchopleural fistula treated with atrial septal defect occluder. Ann Thorac Surg..

[16] Fruchter O, El Raouf BA, Abdel-Rahman N, Saute M, Bruckheimer E, Kramer MR (2014). Efficacy of bronchoscopic closure of a bronchopleural fistula with amplatzer devices: long-term follow-up. Respiration..

[17] Fruchter O, Kramer MR, Dagan T (2011). Endobronchial closure of bronchopleural fistulae using amplatzer devices: our experience and literature review. Chest..

[18] Seder CW, Basu S, Ramsay T (2019). A Prolonged Air Leak Score for Lung Cancer Resection: An Analysis of The Society of Thoracic Surgeons General Thoracic Surgery Database. Ann Thorac Surg..

[19] Clark JM, Cooke DT, Brown LM (2020). Management of Complications After Lung Resection: Prolonged Air Leak and Bronchopleural Fistula. Thorac Surg Clin..

[20] Pairolero PC, Arnold PG, Trastek VF, Meland NB, Kay PP (1990). Postpneumonectomy empyema. The role of intrathoracic muscle transposition. J Thorac Cardiovasc Surg..

[21] Zaheer S, Allen MS, Cassivi SD (2006). Postpneumonectomy empyema: results after the Clagett procedure. Ann Thorac Surg..

[22] Schneiter D, Grodzki T, Lardinois D (2008). Accelerated treatment of postpneumonectomy empyema: a binational long-term study. J Thorac Cardiovasc Surg..

[23] Shekar K, Foot C, Fraser J, Ziegenfuss M, Hopkins P, Windsor M (2010). Bronchopleural fistula: an update for intensivists. J Crit Care..

[24] Pierson DJ, Horton CA, Bates PW (1986). Persistent bronchopleural air leak during mechanical ventilation. A review of 39 cases. Chest..

[25] Motus IY, Bazhenov AV, Basyrov RT, Tsvirenko AS (2020). Endoscopic closure of a bronchopleural fistula after pneumonectomy with the Amplatzer occluder: a step forward?. Interact Cardiovasc Thorac Surg..

